# Urinary Pesticide Levels in Children and Adolescents Residing in Two Agricultural Communities in Mexico

**DOI:** 10.3390/ijerph16040562

**Published:** 2019-02-15

**Authors:** Erick Sierra-Diaz, Alfredo de Jesus Celis-de la Rosa, Felipe Lozano-Kasten, Leonardo Trasande, Alejandro Aarón Peregrina-Lucano, Elena Sandoval-Pinto, Humberto Gonzalez-Chavez

**Affiliations:** 1 Public Health Department, University of Guadalajara, Sierra Mojada 950, Guadalajara, Jalisco CP 44340, Mexico; erksland@hotmail.com (E.S.-D.); f_lozano_k@hotmail.com (F.L.-K.); aapl69@hotmail.com (A.A.P.-L.); elena.sandovalp@academicos.udg.mx (E.S.-P.); 2 Departments of Pediatrics, Environmental Medicine, and Population Health, New York University School of Medicine, New York, NY 10016, USA; leonardo.trasande@nyulangone.org; 3 CIESAS Occidente Conacyt, Av. España 1359, Guadalajara, Jalisco CP 44190, Mexico; hgc@ciesas.edu.mx

**Keywords:** pesticide levels, children, adolescents, agricultural activities, body fluid, exposure

## Abstract

The use of pesticides in agricultural activities has increased significantly during the last decades. Several studies have reported the health damage that results from exposure to pesticides. In Mexico, hundreds of communities depend economically on agricultural activities. The participation of minors in this type of activity and their exposure to pesticides represents a potential public health problem. A cross-sectional study was conducted, in which urine samples (first-morning urine) were taken from children under 15 years of age in both communities. A total of 281 urine samples obtained in both communities were processed for the determination of pesticides with high-performance liquid chromatography together with tandem mass spectrometry. In 100% of the samples, at least two pesticides of the 17 reported in the total samples were detected. The presence of malathion, metoxuron, and glyphosate was remarkable in more than 70% of the cases. Substantial differences were detected regarding the other compounds. It is necessary to carry out long-term studies to determine the damage to health resulting from this constant exposure and to inform the health authorities about the problem in order to implement preventive measures.

## 1. Introduction

According to the World Health Organization (WHO), more than 1000 pesticides are used in agriculture [1]. Each of these chemicals possesses properties and distinct toxicological effects that depend on their function and other factors. Exposure to organic pesticides has been recognized as a growing public health problem, especially in developing countries [2]. In particular, persistent organic compounds used as pesticides are resistant to degradation and are associated with teratogenic and carcinogenic effects on health. Another of their characteristics is their potential for bioaccumulation and biomagnification in foods [3]. In Mexico, by 2017, the State of Jalisco was consolidated as the national leader in agrofood production, participating in 11.48% of the Gross Domestic Product (GDP) of Mexico in the primary sector, the highest in the country. This implies an agricultural extension of 11.7 million hectares [4,5].

An important part of the growth of the agrofood industry derives from the participation of small farming communities that are located throughout the state. As part of the daily activities in many rural Mexican communities, minors are incorporated into the agricultural activities at a very early age, which can represent a health risk due to the continuous and persistent exposure to pesticides. In addition, there is no adequate regulation concerning the sale of pesticides, nor are there training programs for producers and workers to inform them about the risk of exposure for these individuals and their children due to direct contact with pesticides. Thus, in the community, it is common to observe children applying pesticides. Previous research has showed that an organic diet has a positive effect in children, lowering pesticide levels in urine [6].

Another factor that increases exposure is the lack of precautionary measures for the management of pesticides, such as storage of pesticides outside of the home and out of the reach of children, avoidance of the recycling of containers, and the confinement of these to appropriate places.

In one population, in Agua Caliente, Mexico, the reported prevalence of albuminuria in children and adolescents younger than 17 years is over 30%. Exposure to pesticides associated with agricultural activities is a suspected contributor, yet no previous studies have documented the degree of exposure. Therefore, the objective of this study was to measure the concentration and prevalence of pesticides in children and adolescents under 17 years of age and to compare it with those detected in the community of Ahuacapán, which possesses very similar characteristics.

## 2. Material and Methods

A cross-sectional study was carried out simultaneously in two communities. The first of these was, Agua Caliente, near Lake Chapala, the largest lake in Mexico, and the second was a community in the region of the south coast of the state (Ahuacapán).

Beginning hundreds of years ago, multiple autochthonous communities of people of Nahua native origin who were dedicated to fishing and agriculture settled on the bank of Lake Chapala. Since 2016, the Department of Public Health of the University of Guadalajara has carried out studies in the zone, specifically in the community of Agua Caliente, Poncitlán Municipality, State of Jalisco. In this community, health problems have been detected such as malnutrition and albuminuria, specifically in children and adolescents under the age of 17 years [7].

The community is inhabited by 998 persons, whose main activities are farming (37.9%), construction work (29.3%), and laboring 7.2% and who alternate with fishing as a means of subsistence. The most common local crops are corn, seasonal beans, and chayote (*Sechium edule*), which the inhabitants irrigate with lake water. The weekly average family income is approximately 52.63 USD.

The community of Ahuacapán is found toward the coast of the Pacific Ocean, in a 180 km straight line from Agua Caliente. A total of 950 inhabitants live there and their principal economic activity is agriculture, producing sugar cane, corn, tomatoes, citrus, and horticultural products. The water necessary for agriculture derives from springs, deep wells, and the Ayuquila River. The weekly family income in this community is approximately 75 USD. In both localities, there is a total of 550 children and adolescents (58%) aged between less than 1 year and 15 years.

For the urine sampling, we invited the communities to participate voluntarily, with the approval of the Department of Public Health of the University of Guadalajara and the local authorities of the two communities. The sample included only children and adolescents aged under 17 years in Agua Caliente and those under 12 years of age in Ahuacapán. The childrens’ and adolescents’ parents were informed concerning the objective of the study and, after obtaining the parents’ signed consent, the minors were asked for a urine sample (first-morning urine). In both communities, anthropometric measurements (weight and height) were performed on the minors.

Urine samples were transported to the laboratory and were processed for the determination of pesticides with the HPLC/MS/MS (high-performance liquid chromatography coupled with tandem mass spectrometry) method with Agilent Technologies^®^ Model 1200 equipment for HPLC and Model 6430B for MS/MS spectrometry. The method for HPLC used a column Zorbax Eclipse XDB C18, Rapid Resolution, 2.1 × 50 mm, 3.5 µm. Mobile phases: A, 0.1% formic acid in water; B, acetonitrile (ACN); gradient of 40% to 100% B; injection volume, 5 µL; flow, 0.5 min; curve range for each pesticide, 0.01 to 1000 µg/mL [8,9]. The latter was performed at the Laboratory of Applied Pharmacokinetics of the University Center of Exact and Engineering Sciences of the University of Guadalajara. With this method, it was possible to determine the presence of 16 pesticides, which are depicted in Table 1.

**Figure 1:**
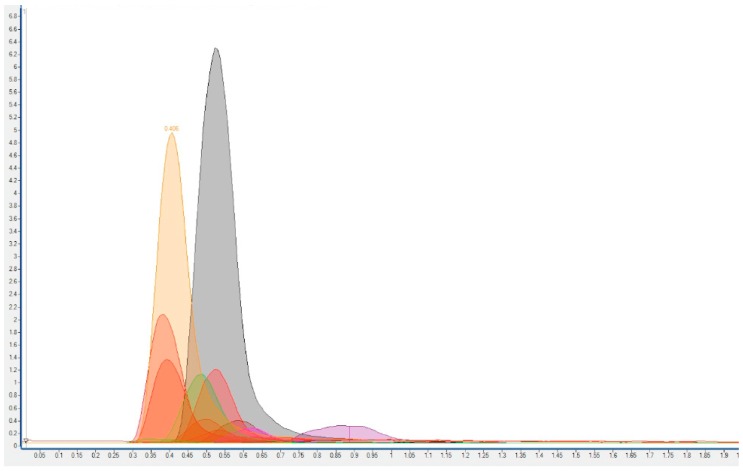
Shows a chromatogram of a urine sample.

Conditions for MS/MS spectrometry are described in Table 2 and Figure 1 shows a chromatogram urine sample.

For the statistical description, we utilized absolute frequencies, percentages, means, and standard deviations (SD). Statistical significance was evaluated by means of the Mann–Whitney *U*, the Chi-squared, and the Fisher exact tests. To compare the two populations and evaluate the differences in the urine pesticide levels, the Mann–Whitney *U* test was used. Similarly, to compare the frequency of detection rate, the Fisher test was used. Statistical significance was considered with a *p* of ≤0.05. For data processing, Excel^®^ (Microsoft, Redmond, WA, USA) and Epi Info ver. 7.2 (Centers for Disease Control and Prevention (CDC) Atlanta, GA, USA) statistical software were used.

This research was carried out with the authorization of the ethics committee of the Department of Public Health of the University of Guadalajara (registration number DCSP/CEI/2016/260618/038).

## 3. Results

A total of 281 children participated, of whom 192 (68.3%) corresponded to the community of Agua Caliente with an average age of 9.4 years (range, 5–15 years). In the community of Ahuacapán, we collected 89 (31.7%) samples and the participants had an average age of 8.31 years (range, 5–13 years; Table 3).

Detection of the pesticides was frequent in both communities. Substantial differences in exposure and detection rate were also identified (Table 4). In general terms, 100% of the study subjects were exposed to at least two of the compounds identified in urine. Positive results are noteworthy of six of the compounds in more than 70% of the subjects studied in both communities: Malathion, metoxuron, glyphosate, dimethoate, enilconazole, and acetochlor.

## 4. Discussion

In the present study, we identified substantial and nearly ubiquitous pesticide exposure in children living in two agricultural communities in Mexico. While no associations with health outcomes were studied, previous research documents harmful effects of these pesticide exposures in pregnant women and children. Neurocognitive effects and occurrence cancer at an early age [10] are not the only consequences of broad use of pesticides with insufficient control. A review of epidemiological studies in 2008 [11] reported that children exposed to pesticides might have difficulties performing tasks that involve short-term memory and impaired mental development.

This definitely does not comprise a minor problem and its extension can be very broad, since in Mexico, the use of pesticides forms part of the daily activities in agricultural communities and Jalisco is one of the three states in Mexico with the greatest index of people poisoned due to the application of pesticides [12].

This work also contributes relevant data on the three categories of the pesticides identified. In this regard, the greatest prevalence was for the herbicides (60.49%), in second place, fungicides (39.05%), and lastly, the insecticides (20.92%). According to our observations in the field, the use of herbicides has intensified, because it allows small and large agricultural enterprises to save considerably on salaries.

On comparing the results obtained in both communities, it is noteworthy that there are differences with respect to prevalence and concentration of pesticides in urine. Clear examples include acetochlor, which was detected in 84% of the minors studied in Agua Caliente vs. 49% in Ahuacapán. In a similar way, and with higher percentages, we found malathion, metoxuron, dimethoate, and enilconazole, with an average detection of 95.7% in Agua Caliente vs. 50% in Ahuacapán (*p* < 0.01). However, we also detected pesticides with a higher frequency in Ahuacapán than in Agua Caliente. Such is the case of glyphosate, which presented in 100% of the minors studied in Ahuacapán vs. 73% in Agua Caliente (*p* > 0.001), with minimal values of 0.0020 µg/mL and maximal values of 2.63 µg/mL. This herbicide was introduced on the market in the 1970s and is currently one of the most utilized herbicides in Mexico and at the global level [13].

Atrazine, carbendazim, and thiabendazole exhibited similar behavior, with a greater frequency in Ahuacapán and with an average percentage of 33% vs. 13.8% (*p* < 0.01). Herbicides, as well as fungicides, are widely employed by the sugar cane and horticultural agro-industries in Ahuacapán. In this town, there are also small producers of basic crops such as corn, beans, squash, and chilies, on which herbicides are also widely utilized. Thus, the differences between the two localities are associated with the agro-industrial activity and the practices that have become generalized among the small producers of basic crops. In Agua Caliente, the town’s proximity to Lake Chapala, with the latter forming part of one of the most contaminated hydrological basins of Mexico, doubtlessly plays an outstanding role [14].

One of the main sources of exposure is the environment. Many pesticides have the capacity to transfer from one matrix to another. Once the fumigation is performed, the residues are deposited in the soil and, through infiltration processes, the compounds are leached out of the soil by the rain until they reach bodies of water, with the consequent transfer to aquatic organisms, or they can eventually reach phreatic (groundwater) levels, where they can be extracted through wells for human use [12].

In 2015, Bradman et al. reported an interventional study in order to compare the pesticides in urine in 20 children from an urban zone vs. 20 children from a rural zone in California, U.S. The participating children were aged ranging from 3–6 years (19 boys and 21 girls). A feeding protocol was established that included four days of a conventional diet, seven days of an organic diet, and five additional days of a conventional diet. The results reported that 2,4-Dichlorophenoxyacetic acid and metolachlor were found in 90% and 72% of the urine samples. The authors concluded that exposure to organophosphates (dialkyl phosphates and dimethyls) is lower in children with an organic diet, while the other frequently detected pesticides (pyrethroids, diethyl organophosphate pesticides, and metolachlor) did not diminish in a significant manner during the organic-diet phase [6]. With respect to the 2,4-Dichlorophenoxyacetic acid, in our study, this was measured only in one community (Agua Caliente) and was solely detected in 27% of the minors studied.

The results obtained by Bradman in 2015 [6] are consistent with studies reported by Lu et al. who, in 2008, reported that in a group of 23 children ranging in age between 3 and 11 years, that the concentrations of organophosphates, malathion, and chlorpyrifos in urine diminished when a conventional diet was changed to a feeding regimen with organic products [15].

The protocol utilized in both studies yields valuable evidence with respect to exposure to pesticides in pediatric populations through the consumption of non-organic foods. Our sample only reveals the result of a sole urine sample during the winter, which is when there is a significant diminution in the agricultural use of pesticides. However, consumption of a conventional diet with seasonal products could play a very important role regarding the presence of these pesticides in urine.

The international literature describes that the use of organophosphates is extensive at the worldwide level, and that, due to their toxicity, they represent a serious public health problem in developing countries [16]. Although it is not an initial objective of this study, it is important to take the data obtained into account, since the high frequency of these pesticides detected in urine could signify potential damage to the health of the minors studied in both communities.

In 2010, Hernández and Hassen carried out a study in agricultural zones of the Mexican state of Sonora. Despite restriction of the use of DDT (dichlorodiphenyltrichloroethane) in agriculture in Mexico (Official Journal of the Federation 1991), these authors reported that this pesticide was found in environmental samples due to its long half-life. By means of the sampling and analysis of water and sediments, the authors evaluated the environmental impact due to the use of these chemicals. Finally, they suggested that the presence of pesticides in concentrations above the established limits should involve surveillance and monitoring in bodies of water [17].

Our study generates very important initial information for taking future measures, in addition to attracting the attention of the health authorities. Despite one of the weaknesses of the study being that the results were generated through a single urine sample, it presents to us the opportunity of establishing investigation projects that permit us to identify whether there are changes in the levels of the pesticides based on the different seasons of the year and to consider, in greater detail, whether there is a relationship between the presence of pesticides and the change in the patterns of agricultural production. The University of Guadalajara conducted this research at this time.

Continuous monitoring is of vital importance in order to identify health problems that already could be present and to achieve preventive strategies to avoid as much as possible the participation of minors in agricultural activities. In addition, we consider it convenient to implement educative workshops that orient the population concerning the risks to health associated with management and exposure to pesticides. The health authorities at the regional and federal levels in Mexico should carry out this activity, but they have ignored their responsibility. The University of Guadalajara in 2016 conducted a census that reported that 94% of people engaged in agricultural activities apply pesticides. To date, to our knowledge, there are no reports regarding whether the population knows the risks of this exposure and whether they are trained in the application of chemicals for agricultural use.

## 5. Conclusions

We identified frequent and substantial detection of multiple pesticides in the urine of children living in two agricultural communities in Mexico, Agua Caliente and Ahuacapán. Future studies will allow us to understand the potential consequences of these exposures for kidney function in youth.

## Figures and Tables

**Table 1:** Category of pesticides analyzed in urine samples from the two communities.

Name	IUPAC ID	PubChem CID	Agrochemical Category
Acetochlor	2-chloro-N-(ethoxymethyl)-N- (2-ethyl-6-methylphenyl)acetamide	1988	Herbicide
Atrazine	6-chloro-4-N-ethyl-2-N-propan-2-yl-1,3,5-triazine-2,4-diamine	2256	Herbicide
Carbendazim	methyl N-(1H-benzimidazol-2-yl)carbamate	25429	Fungicide
Carbofuran	(2,2-dimethyl-3H-1-benzofuran-7-yl) N-methylcarbamate	2566	Insecticide, Nematicide, Acaricide
Cyhalothrin	[cyano-(3-phenoxyphenyl)methyl] 3-[(Z)-2-chloro-3,3,3-trifluoroprop-1-enyl]-2,2-dimethylcyclopropane-1-carboxylate	5281873	Insecticide
Diazinon	*O*,*O*-Diethyl O-[4-methyl-6-(propan-2-yl) pyrimidin-2-yl] phosphorothioate	3017	Insecticide, Acaricide
Dimethoate	2-dimethoxyphosphinothioylsulfanyl-N-methylacetamide	3082	Insecticide, Acaricide
Emamectin	4″-Deoxy-4″-epi-methylamino-avermectin B1; Epi-methylamino-4″-deoxy-avermectin	11549937	Insecticide
Enilconazole (imazalil)	1-[2-(2,4-dichlorophenyl)-2-prop-2-enoxyethyl]imidazole	37175	Fungicide
Glyphosate	2-(phosphonomethylamino)acetic acid	3496	Herbicide
Malathion	diethyl 2-dimethoxyphosphinothioylsulfanylbutanedioate	4004	Insecticide, Acaricide
Methomyl	methyl (1E)-N-(methylcarbamoyloxy)ethanimidothioate	5353758	Insecticide
Metoxuron	3-(3-chloro-4-methoxyphenyl)-1,1-dimethylurea	29863	Herbicide
Molinate	S-ethyl azepane-1-carbothioate	16653	Herbicide
Pyraclostrobin	Methyl N-[2-[[1-(4-chlorophenyl) pyrazol-3-yl]oxymethyl]phenyl]-N-methoxycarbamate	6422843	Fungicide, plant growth regulator
Thiabendazole	4-(1H-benzimidazol-2-yl)-1,3-thiazole	5430	Fungicide

**Table 2:** Mass spectrometer conditions for pesticide determination.

Mass spectrometer conditions				
Electrospray Interface Condition				
Gas emperature	350 °C			
Gas flow	12 L/min			
Nebulizer	25 psi			
Capillary	+4000	−4000		
Compound name	**Precursor Ion**	**Product Ion**	**Fragmentor**	**Polarity**
L-Cyhalotrin (225.1)	467.1	225.1	80	Positive
Meclizina (201.1)	391.2	201.1	90	Positive
Pyraclostrobin (163)	388	163	120	Positive
Malation (99)	331	99	80	Positive
Clorpyrifos (200)	325	200	30	Positive
Oxandrolona (289.2)	307.2	289.2	100	Positive
Oxandrolona (271.2)	307.2	271.2	100	Positive
Oxandrolona (229.1)	307.2	229.1	100	Positive
Diazinon (153)	305	153	160	Positive
Imazalil (159)	297	159	160	Positive
Paration (264)	292	264	90	Positive
Paration (236)	292	236	90	Positive
Acetoclor (224.2)	270.1	224.2	60	Positive
Acetoclor (148.4)	270.1	148.4	60	Positive
Picloram (222.9)	240.9	222.9	90	Positive
Picloram (194.9)	240.9	194.9	90	Positive
Dimethoate (171)	230	171	80	Positive
Metoxuron (72.1)	229.1	72.1	93	Positive
Ametryn (186)	228.1	186	120	Positive
Ametryn (96)	228.1	96	120	Positive
Carbofuran (123)	222	123	120	Positive
Atrazine (132)	216	132	120	Positive
Thiabendazole (131)	202	131	120	Positive
Carbendazim (160)	192.1	160	110	Positive
Molinate (55.1)	188.1	55.1	78	Positive
Methomyl (106)	163.1	106	30	Positive
Methomyl (88.1)	163.1	88.1	30	Positive
Methomyl (65)	163.1	65	30	Positive
Emamectina (158.1)	887.1	158.1	60	Positive
Glyphosate (149.9)	168	149.9	80	Negative
Glyphosate (124.2)	168	124.2	80	Negative
2,4-D (161.1)	219	161.1	50	Negative

**Table 3:** Demographic and anthropometric data of the children in both communities.

Variable	Agua Caliente (*n* = 192)	Ahuacapán (*n* = 89)
Gender		
Female	84 (43.8%)	40 (44.9%)
Male	108 (56.3%)	49 (55.1%)
Age (years)	9.40 (SD 2.52)	9.31 (SD 2.05)
Age groups		
5–8 y	78 (40.6%)	49 (55.1%)
9–11 y	67 (34.9%)	34 (38.21%)
12–15 y	47 (24.5%)	6 (6.7%)
Weight (kilograms)	29.39 (SD 10.06)	32.27 (SD 11.73)
Height (centimeters)	131.58 (SD 14.12)	132.94 (SD12.97)
Body mass index (k/m^2^)	16.46 (SD 2.44)	17.72 (SD 3.66)

**Table 4:** Frequencies, percentages, means, and standard deviations (SD) of pesticides in urine.

Pesticide	Agua Caliente	Ahuacapán	
	*n* (%)	*n* (%)	*p* (Fisher Test)
	Mean µg/mL (SD)	Mean µg/mL (SD)	*p* (Mann–Whitney)
**Acetochlor**	161 (83.85)	44 (49.43)	<0.01
	0.008 (0.0867)	0.001 (0.0017)	0.04
**Atrazine**	22 (11.45)	22 (24.71)	<0.01
	0.016 (0.0486)	0.043 (0.0930)	0.06
**Carbendazim**	29 (15.10)	52 (41.57)	<0.01
	0.141 (0.4192)	0.330 (0.5040)	<0.01
**Carbofuran**	1 (0.52)	0	NA
	0.246		NA
**Cyhalothrin**	138 (71.87)	45 (50.56)	<0.01
	0.083 (0.0823)	0.080 (0.0855)	0.52
**Diazinon**	29 (15.10)	20 (22.47)	0.09
	0.007 (0.0199)	0.008 ± 0.0180	0.41
**Dimethoate**	179 (93.22)	44 (49.43)	<0.01
	0.146 (0.1834)	0.169 (0.2299)	0.03
**Emamectin**	6 (3.12)	9 (10.11)	0.02
	0.006 (0.0339)	0.019 (0.0582)	0.34
**Enilconazole**	177 (92.18)	29 (32.58)	<0.01
	1.582 (5.6623)	0.069 (0.1023)	<0.01
**Glyphosate**	140 (72.91)	89 (100)	<0.01
	0.363 (0.3210)	0.6060 (0.5435)	<0.01
**Malathion**	191 (99.47)	55 (61.79)	<0.01
	0.681 (0.6431)	0.177 (0.1730)	<0.01
**Methomyl**	46 (23.95)	0	<0.01
	0.016 (0.0292)		<0.01
**Metoxuron**	188 (97.91)	50 (56.17)	<0.01
	0.038 (0.0403)	0.037 (0.0414)	0.10
**Molinate**	79 (41.14)	55 (61.79)	<0.01
	0.191 (0.3698)	0.273 (0.4240)	<0.01
**Pyraclostrobin**	62 (32.29)	37 (41.57)	0.08
	0.049 (0.2331)	0.042 (0.0509)	0.18
**Thiabendazole**	29 (15.10)	24 (26.96)	<0.01
	0.007 (0.0511)	0.002 (0.0046)	0.12
