# Erratum: Sierra-Diaz, E.; Celis-de la Rosa, A.J.; Lozano-Kasten, F.; Trasande, L.; Peregrina-Lucano, A.A.; Sandoval-Pinto, E.; Gonzalez-Chavez, H. Urinary Pesticide Levels in Children and Adolescents Residing in Two Agricultural Communities in Mexico. *Int. J. Environ. Res. Public Health* 2019, *16*, 562

**DOI:** 10.3390/ijerph17010159

**Published:** 2019-12-24

**Authors:** Erick Sierra-Diaz, Alfredo de Jesus Celis-de la Rosa, Felipe Lozano-Kasten, Leonardo Trasande, Alejandro Aarón Peregrina-Lucano, Elena Sandoval-Pinto, Humberto Gonzalez-Chavez

**Affiliations:** 1 Public Health Department, University of Guadalajara, Sierra Mojada 950, Guadalajara, Jalisco CP 44340, Mexico; erksland@hotmail.com (E.S.-D.); f_lozano_k@hotmail.com (F.L.-K.); aapl69@hotmail.com (A.A.P.-L.); elena.sandovalp@academicos.udg.mx (E.S.-P.); 2 Departments of Pediatrics, Environmental Medicine, and Population Health, New York University School of Medicine, New York, NY 10016, USA; leonardo.trasande@nyulangone.org; 3 CIESAS Occidente Conacyt, Av. España 1359, Guadalajara, Jalisco CP 44190, Mexico; hgc@ciesas.edu.mx

The authors would like to update some important data in the manuscript. In Table 4, the pesticide means were reported in µg/mL, which is incorrect. The correct units are ng/mL (nanograms/milliliter). The same typographical inaccuracy applies for data in the fourth paragraph of the discussion (with minimal values of 0.0020 µg/mL and maximal values of 2.63 µg/mL), where the correct units are also ng/mL [1].

We want to mention that this typographical oversight does not result in any changes to the conclusions or the main subject of the research, since the data focuses on the prevalence of pesticides. It is important for us to report on this concern in order to avoid readers misinterpreting the results.
